# The coronary reimplantation after neoaortic reconstruction technique can make a difference in arterial switch operation

**DOI:** 10.1186/s13019-019-0994-8

**Published:** 2019-09-18

**Authors:** Kwang Ho Choi, Si Chan Sung, Hyungtae Kim, Hyoung Doo Lee, Hoon Ko, Joung-Hee Byun

**Affiliations:** 1 Department of Thoracic and Cardiovascular Surgery, Research Institute for Convergence of Biomedical Science and Technology, Pusan National University College of Medicine, Pusan National University Yangsan Hospital, Geumo-ro 20, Beomeo-ri, Mulgeum-eup, Yangsan-si, Gyeongsangnam-do 50612 South Korea; 2 Department of Pediatrics, Research Institute for Convergence of Biomedical Science and Technology, Pusan National University College of Medicine, Pusan National University Yangsan Hospital, Yangsan-si, Gyeongsangnam-do South Korea

**Keywords:** Arterial switch operation, Coronary artery reimplantation, Neoaortic reconstruction

## Abstract

**Background:**

The aim of this study was to determine if there was a difference between coronary reimplantation after neoaortic reconstruction and open coronary reimplantation technique in arterial switch operation (ASO).

**Methods:**

A total of 236 patients who underwent ASO from March 1994 to August 2018 were enrolled in this study. Multivariate analysis was performed for postoperative early mortality. Patients were divided into the open coronary reimplantation and coronary reimplantation after neoaortic reconstruction groups. The 30-day mortality, intraoperative and postoperative coronary artery (CA) revisions, CA–related late morbidity and mortality, and early and late neoaortic valve regurgitations after ASO were compared between the two groups.

**Results:**

Overall postoperative early mortality was 7.2% (17/236). Patients who underwent open coronary reimplantation had higher early mortality as compared with those who underwent coronary reimplantation after neoaortic reconstruction. Risk factors for postoperative early mortality from multivariate analysis were cardiopulmonary bypass time and open coronary reimplantation. There was a higher incidence of CA–related late mortality or morbidity in the open coronary reimplantation group. The open coronary reimplantation group had a higher incidence of intraoperative or postoperative CA revision. There were no differences in the incidence of mild or more neoaortic valve regurgitation at discharge or in the 5-year freedom from mild or more neoaortic valve regurgitation.

**Conclusions:**

CA reimplantation after neoaortic reconstruction yields better results in mortality and intraoperative or postoperative CA–related problems in ASO without increasing postoperative neoaortic valve regurgitation.

## Background

Coronary implantation is one of the most critical process in arterial switch operation (ASO). Originally Jatene et al. [1] used coronary artery (CA) reimplantation after neoaortic reconstruction in their first ASO. Thereafter, Pacifico et al. [2] and Bove [3] reported the same technique in 1983 and 1989, respectively. The main advantage of this technique is that surgeons can easily identify the accurate site of the CA transfer regardless of CA anatomy, alignment of the facing commissure, and level of CA origin, which can reduce CA transfer related problems. We reported better results of coronary reimplantation after neoaortic reconstruction (CRANR) in 2005 [4]. However, many pediatric cardiac surgeons are currently using the open coronary reimplanation (OCR) technique. We herein evaluated the outcome of the ASO and compared the outcome of the two techniques in terms of 30-day operative mortality, CA-related early and late problems, and neoaortic valve regurgitation to determine if there was a difference between CRANR and OCR.

## Methods

**Fig. 1:**
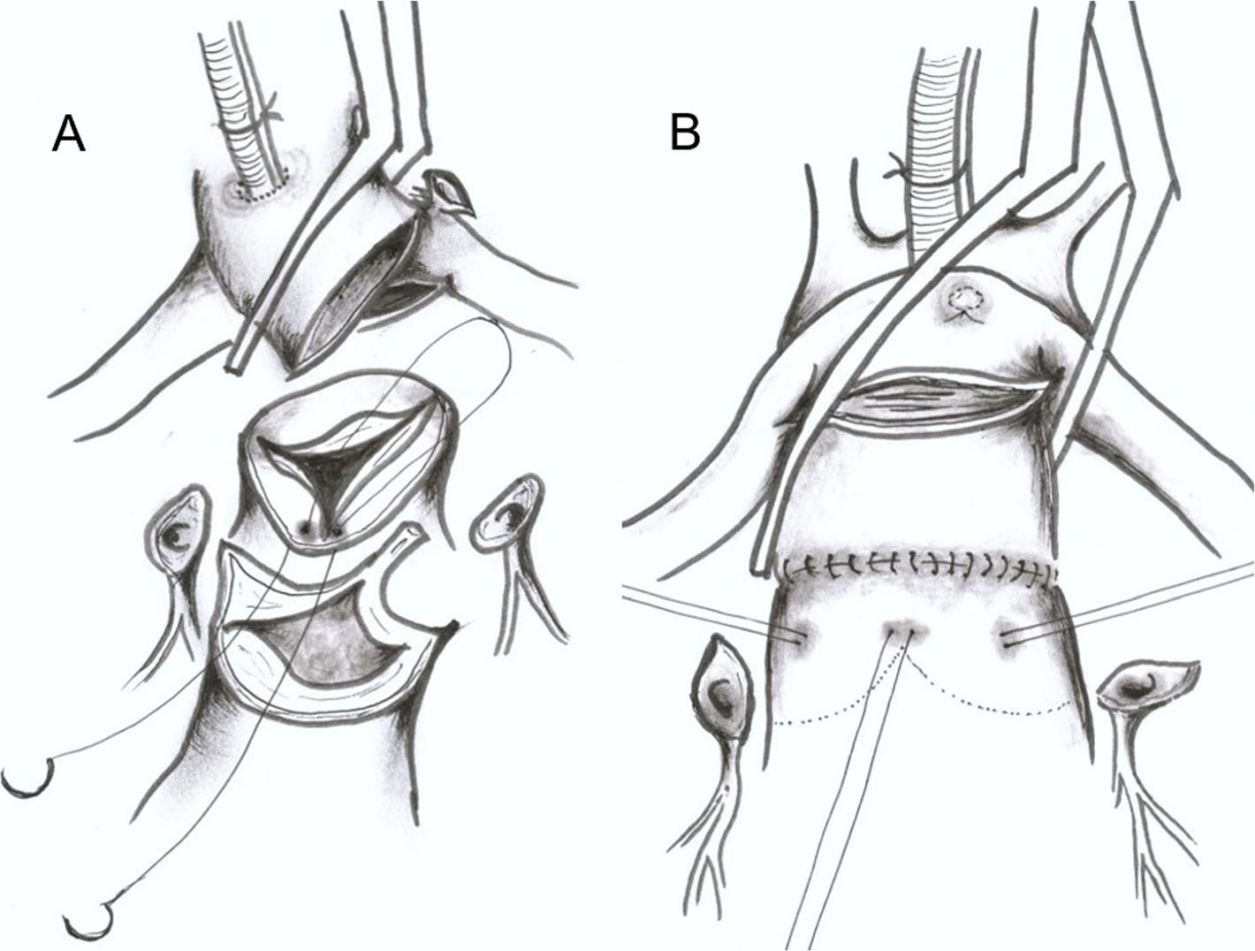
Operative technique: (**a**) Making the marking stitches; the site of the commissural attachment between facing sinuses as inserting needles from inside to outside. (**b**) Identification of the exact location for coronary reimplantation after aortic cross-clamp was released

**Fig. 2:**
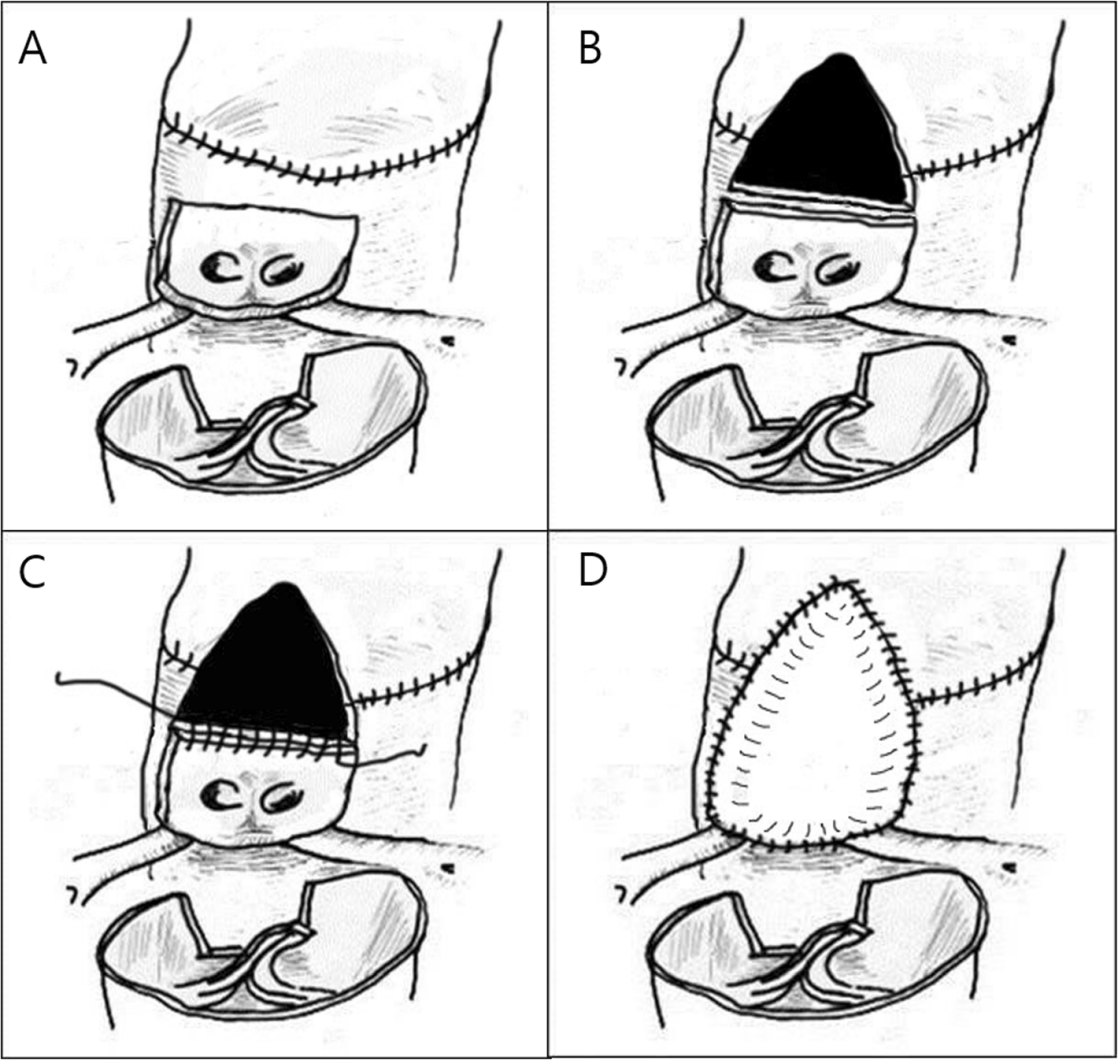
Coronary reimplantation after neoaortic reconstruction in the aortocoronary flap technique: (**a**) commissural detachment and creation of coronary artery (CA) button following neoaorta reconstruction. (**b**) Placement of the CA button on the distended neoaorta and creation of a large window just above sinotubular junction. (**c**) Creation of an anastomosis between the upper border of the CA button and the lower border of the window. (**d**) Redundant covering of CA button and window with autologous pericardial patch

Institutional review board approvals were obtained for this study. Between March 1994 to Aug. 2018, 236 patients underwent ASO in Donga University Hospital and Pusan National University Hospital. All operations were performed by the same group of surgeons. Preoperative diagnoses were transposition of the great arteries (TGA) with intact ventricular septum (*n* = 120), TGA with ventricular septal defect (VSD) (*n* = 63), TGA with VSD and arch anomaly (*n* = 10), Taussig-Bing anomaly (*n* = 15), and Taussig-Bing anomaly with arch anomaly (*n* = 28). The different types of coronary anatomies are listed in Table 1. No important changes in preoperative, intraoperative, and postoperative managements were established during this study period, including myocardial protection and cardiopulmonary bypass (CPB) strategy. The operative technique was described in detail in our previous report [4], but the coronary transfer technique is summarized here. In the OCR technique, after creation of the coronary buttons, the main pulmonary artery (MPA) was cut at the site about 2 mm proximal to its bifurcation. The trap door incisions were made on the divided proximal MPA at the site of the marking stitches, which were placed before cannulation, and the coronary buttons were implanted to the incisions. This proximal MPA with coronary buttons was then anastomosed to the distal ascending aorta after Lecompte maneuver. This CA transfer technique was used in 72 patients (OCR group). In the CRANR technique, the neoaorta was reconstructed before CA transfer. The distal ascending aorta was anastomosed to the divided proximal MPA after the Lecompte maneuver. Once the neoaortic anastomosis was completed, the aortic cross-clamp (ACC) was temporarily released, allowing the neoaorta to distend in order to facilitate the identification of the exact location for coronary reimplantation. The ideal locations on the neoaortic root were marked with two traction stay stitches on each side. The ACC was then reapplied, and two centrally tilting incisions were made between the stay stitches for coronary button implantations. We sometimes added a small trapdoor incision to prevent excessive torsion of the CA. We usually made large C-shaped or reverse C-shaped trapdoor incision for the single CA. The coronary buttons were then transferred to the neoaorta. In this technique, we did not create marking stitches on the neoaorta (MPA) to select the location for coronary reimplantation before cannulations. Instead, we made a marking stitch at the site of the facing commissure by inserting needles from inside to outside using a double-armed fine suture. This maneuver is very important for locating the commissural site of the neoaorta from the outside after neoaortic reconstruction (Fig. 1). This CRANR technique was used in 164 patients (CRANR group). We have adopted the CRANR technique since 2004 in individual CA transfer. However, in the aortocoronary flap technique, the CRANR technique has only been used since 2010 (Fig. 2). Among 14 patients who underwent the aortocoronary flap procedure, the OCR technique was performed in 11 patients and CRANR was perfromed in the remaining 3 patients.

**Table 1:** Characteristics of the patients

Variables	OCR (*n* = 72)	CRANR (*n* = 164)	*p*-value
Male	57 (79.2)	112 (68.3)	0.088
Age (days)	23.4 ± 28.5	12.1 ± 16.5	0.002
Body weight (kg,)	3.5 ± 0.7	3.4 ± 0.5	0.055
Diagnosis			0.136
TGA-IVS	35 (48.6)	85 (51.8)	
TGA-VSD	20 (27.8)	43 (26.2)	
TGA-VSD-AA	6 (8.3)	4 (2.4)	
TB	6 (8.3)	9 (5.5)	
TB-AA	5 (6.9)	23 (14.0)	
GA relationship (AP)	60 (83.3)	123 (76.9)	0.265
AA	11 (15.3)	27 (16.5)	0.820
VSD	37 (51.4)	79 (48.2)	0.649
CA			
Usual	44 (61.1)	110 (67.1)	0.376
Unusual	28 (38.9)	54 (32.9)	0.376
Intramural	9 (12.5)	7 (4.3)	0.021
Retropulmonary	15 (20.8)	40 (24.4)	0.552
CPB time (min)	243.4 ± 64.6	223.6 ± 47.8	0.022
ACC time (min)	129.2 ± 38.7	138.6 ± 31.8	0.053

Values are presented as mean ± standard deviation or n (%)

*AA* arch anomaly, *ACC* aortic cross clamp, *AP* antero-posterior, *CA* coronary artery, *CPB* cardiopulmonary bypass, *CRANR* coronary reimplantation after neoaortic reconstruction, *GA* great artery, *OCR* open coronary reimplantation, *TB* Taussig-Bing anomaly, *TB-AA* Taussig-Bing anomaly with arch anomaly, *TGA-IVS* transposition of the great arteries with intact ventricular septum, *TGA-VSD* transposition of the great arteries with ventricular septal defect, *TGA-VSD-AA* transposition of the great arteries with ventricular septal defect and arch anomaly, *VSD* ventricular septal defect

For the intramural CA, we have changed the CA transfer technique from aortocoronary flap to individual coronary implantation with long segment unroofing since October 2007. As a result, we used OCR in 9 patients and CRANR in 7. We evaluated the 30-day mortality, incidence of intraoperative and postoperative CA revisions, CA-related morbidity and late death, and incidence of mild or more neoaortic valve regurgitation at discharge and at follow-up through a retrospective study. We defined CA revision when reinstitution of CPB was required. Placement of simple traction was excluded. We evaluated the risk factors of 30-day mortality through multivariate analysis of several variables and compared the incidence of the intraoperative and postoperative CA revisions, CA-related late morbidity and mortality, and early and late postoperative mild or more neoaortic valve regurgitations between the two groups.

### Statistical analysis

Data analyses were performed with SPSS version 19.0 (IBM Corp., Armonk, NY, USA). Continuous data are presented as means with standard deviation. Frequencies are presented as absolute numbers and percentages. The Chi-square test or Fisher exact test were used to compare categorical variables and the t-tests were used to compare continuous variables. The Kaplan-Meier method was used to estimate freedom from significant aortic valve regurgitation and Log-rank test for compare two groups. A *p*-value of < 0.05 was set as the level of statistical significance.

## Results

There were no significant differences between the two groups in terms of sex, body weight, diagnosis, incidence of overall unusual CA, great artery relationship, arch anomalies, and incidence of VSD. However, there were differences in age at operation, the incidence of intramural CA, and CPB time (Table 1).

### Thirty-day mortality

Overall postoperative early mortality was 7.2% (17/236). There was no difference in mortality between patients with usual and unusual coronary patterns [7.8% (12/154) vs. 6.1% (5/82); *p* = 0.632]. There was no difference between patients with intramural and non-intramural CA patterns [6.2% (1/16) vs. 7.3% (16/220); *p* = 0.677]. Patients with arch anomaly had higher early mortality [21.1% (8/38) vs. 4.5% (9/198); *p* < 0.001]. The 30-day mortality was higher in the OCR group than in the CRANR group [19.4% (14/72) vs. 1.8% (3/164); *p* < 0.001]. The 30-day mortality in patients with aortic arch anomalies was also higher in the OCR group [54.5% (6/11) vs. 7.4% (2/27); *p* = 0.004]. Multivariate analysis revealed that CPB time and OCR were risk factors for 30-day mortality (Table 2).

**Table 2:** Logistic regression analysis

Variables	Univariate		Multivariate	
	*p*-value	OR (95% CI)	*p*-value	OR (95% CI)
Female sex	0.514	1.413 (0.501, 3.988)		
Body weight	0.218	0.551 (0.214, 1.421)		
Age	0.492	1.006 (0.988, 1.025)		
VSD	0.027	3.660 (1.157, 11.579)	0.672	0.709 (0.145, 3.470)
Arch anomaly	0.001	5.600 (2.005, 15.644)	0.071	4.265 (0.883, 20.608)
Unusual CA	0.632	0.768 (0.261, 2.262)		
Retropulmonary CA	0.982	1.014 (0.317, 3.245)		
Single coronary sinus	0.337	0.365 (0.047, 2.851)		
Intramural CA	0.879	0.850 (0.105, 6.853)		
OCR	< 0.001	12.954 (3.592, 46.711)	< 0.001	16.697 (3.626, 76.886)
CPB	< 0.001	1.023 (1.013, 1.032)	< 0.001	1.021 (1.009, 1.032)
ACC	0.313	1.007 (0.994, 1.020)		

*ACC* aortic cross clamp, *CA* coronary artery, *CI* confidence interval, *OCR* open coronary reimplantation, *OR* odds ratio, *VSD* ventricular septal defect

### Intraoperative or postoperative CA revisions

Four patients (5.6%) in the OCR group required intraoperative CA revision, but only one patient (0.6%) in the CRANR group required it. One patient with left intramural CA who underwent aortocoronary flap operation using the OCR technique required CA revision 3 days after the operation. After this event, we did not use the aortocoronary flap technique for intramural CA transfer anymore. We have been using individual CA transfer technique since then. The only patient who required intraoperative CA revision in the CRANR group had left intramural CA. This case was our early experience in ASO, and the patient survived the operation after left subclavian artery free grafting to the left CA. There was no late CA revision after the operation in either group. The total incidence of intraoperative and postoperative CA revision was 2.1% (5/236): 5.6% (4/72) in the OCR group and 0.6% (1/164) in the CRANR group (*p* = 0.031).

### CA-related late mortality and morbidity

Overall late mortality beyond 30 days after operation occurred in 10 patients; with 7 patients (12.1%, 7/ 58) in the OCR group and 3 patients (1.9%, 3/ 161) in the CRANR group. Among these, CA-related deaths including all sudden death were 3 in the OCR group and none in the CRANR group. One patient who underwent left subclavian free grafting as salvage procedure after aortocoronary flap procedure using OCR technique required CA stenting 6.5 years after ASO and cardiac transplantation at age of 18 years due to cardiomyopathy. There was no CA-related reoperation or reintervention required in the CRANR group. Late mortality, or morbidity related coronary problems occurred in 4 patients (6.9%, 4/58) in the OCR group, but there was none in the CRANR group.

### Postoperative neoaortic regurgitation

**Fig. 3:**
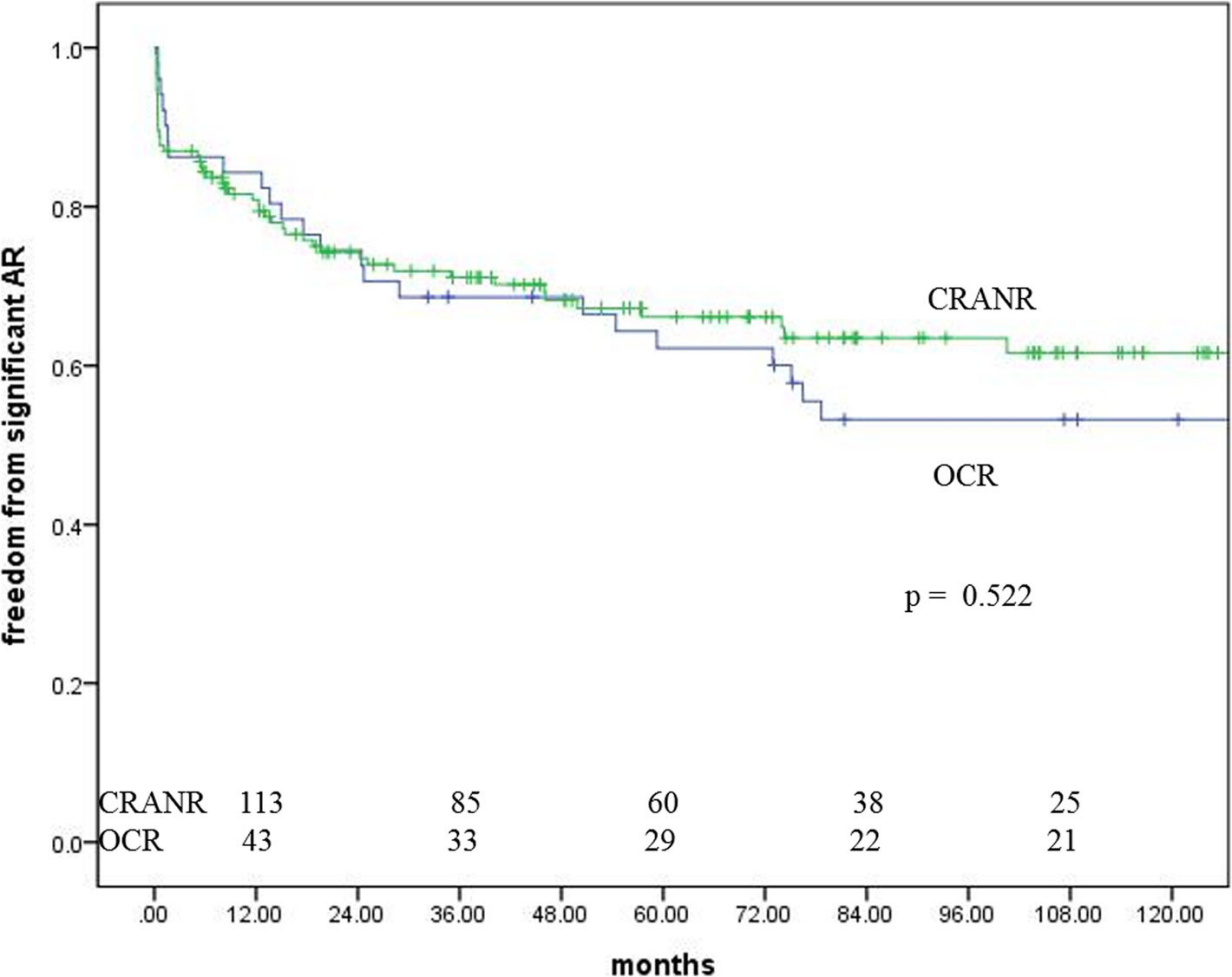
Freedom from significant aortic regurgitation. AR, aortic regurgitation; CRANR, coronary reimplantation after neoaortic reconstruction; OCR, open coronary reimplantation

Postoperative mild or more neoaortic valve regurgitation was observed in 33 patients at discharge: 9 (17.0%) in the OCR group and 24 (15.3%) in the CRANR group. There was no statistical difference in the incidence of neoaortic valve regurgitation at discharge (*p* = 0.769). The freedom from mild or more neoaortic valve regurgitation was 84.3, 68.6 and 62.2% in the OCR group and 80.9, 71.1 and 66.1% in the CRANR group at 1, 3, and 5 years, respectively (Fig. 3). There was no significant difference in freedom from mild or more neoaortic valve regurgitation (*p* = 0.522). The CRANR group did not show any higher incidence of postoperative neoaortic valve regurgitation at discharge or long after the operation. Only two patients, who were in the CRANR group, had moderate neoaortic valve regurgitation in our series. None underwent reoperation for neoaortic valve regurgitation in either group.

## Discussion

ASO is still a challenging operation for less experienced surgeons, even though it has been performed with very low mortality in many high-volume centers. Accurate transfer of the CA is the key component to a successful ASO. Inadequate coronary transfer can lead to early postoperative mortality or late CA-related morbidity.

There are basically two different coronary transfer techniques: open coronary transfer before neoaortic reconstruction (OCR technique) and coronary transfer after neoaortic reconstruction (CRANR technique). The open trap door technique was advocated by Yacoub and Radley-Smith [5] and Brawn and Mee [6]. This coronary transfer technique has been used in many centers, with low operative mortality thus far. In this technique, marking stitches are made at the MPA before inducing cardiac arrest to select accurate sites for CA reimplantation. However, it is not easy for the less experienced surgeons to select the accurate site for coronary reimplantation and it might be further difficult in patients with malaligned commissures or with high take-off CAs. Furthermore, a transferred CA can be distorted during neoaortic reconstruction. The CRANR technique can offer surgeons an easy way for selecting an accurate CA transfer site regardless of malalignment of the facing commissures or abnormal take-off of the CA. Neoaortic reconstruction does not affect CA geometry. The CRANR technique allows easier and more accurate coronary transfer even in the aortocoronary flap technique (Fig. 1c). In the aortocoronary flap procedure, accurate placement of the flap is very important because a small derangement of the flap can cause distortion of one of the two CAs. The CRANR technique allows more accurate placement of the flap at the distended neoaorta. We have adopted the CRANR technique in coronary transfer using aortocoronary flap since September, 2010, when we experienced myocardial ischemia in a patient with intramural CA who underwent aortocoronary flap procedure through OCR. The only disadvantage of the CRANR technique is the possibility of damage in the neoaortic valve when making incisions at the closed neoaorta for coronary transfer. We have overcome this problem by making a marking stitch at the site of the anterior commissural attachment of the neoaorta by inserting needles from inside to outside using a double-armed fine suture before neoaortic reconstruction [4]. This technique was very useful in locating the commissural site of the neoaorta from the outside after neoaorta reconstruction.

We have already reported the differences in the early results between the two techniques in 2005 [4]. In the present study, we added our accumulated data, including those of the aortocronary flap procedure, to the previous data and we also described the long-term data on late coronary problem and neoaortic valve regurgitation.

We performed multivariate analysis for 30-day mortality after ASO. The CPB time and OCR were the risk factors for the 30-day mortality. The odd ratio of the OCR was 16.697. The 30-day mortality in the OCR group was unacceptably high even though the learning curve was taken into account. However, the number of patients with 30-day mortality after ASO using the CRANR technique (3/164, 1.8%) is comparable with the results of previous studies [7, 8]. Among three mortalities, two occurred in patients with aortic arch anomaly. There was only one mortality in ASO without aortic arch repair. We also observed lower 30-day mortality rate in the CRANR group as compared with the OCR group in the patients with aortic arch anomaly. There was no 30-day mortality after ASO in the recent 10 years in this whole series. There has been no report comparing the mortality between OCR and CRANR technique thus far.

Regarding intraoperative and postoperative CA revision, the incidence was higher in the OCR group. In this series, we defined “CA revision” as revision under another CPB. Placement of a simple traction suture to ameliorate distortion of the implanted CA was excluded. In the CRANR group, only one patient with intramural CA, required intraoperative CA revision. We attempted to use individual CA implantation technique for this patient and observed intraoperative ischemia on the left ventricular territory. We placed the left subclavian artery free graft between the left main CA and the ascending aorta. The patient survived the operation. Excluding this patient, there was no intraoperative or postoperative CA revision required in the CRANR group. This means that the CRANR technique is a safe procedure for coronary reimplantation in ASO.

Several studies have reported obstructed CAs in 5 to 7% of survivors [8–12]. Late coronary obstruction after ASO is uncommon and is more frequently anatomic problem. The incidence of CA-related problem is most prevalent in the first 3 months after ASO [13]. One study showed 88.1% ± 6.4% freedom from coronary events at 22 years [14]. We reviewed CA-related late mortality or morbidity in our series. All sudden deaths were assumed to be CA-related deaths because it is very difficult to obtain autopsy data from such patients in our country. There was no sudden death or CA-related late morbidity in the CRANR group. We do not have any routinely scheduled CA evaluation program after ASO. If there is any sign of abnormal echocardiographic findings or cardiac marker elevation, coronary angiography is performed. There has been no late CA-related problem in the CRANR group thus far. Meanwhile, the OCR group not only had higher incidence of intraoperative or postoperative CA revision [5/72 (6.9%) vs 1/164 (0.6%); *p* = 0.011] but also had higher incidence of CA-related late mortality or morbidity in the OCR group [6.9% (4/58) vs 0% (0/161); *p* = 0.005]. We observed three cases of sudden death in the OCR group but none in the CRANR group.

Considering the incidence of mild or more neoaortic valve regurgitation after ASO, previous studies showed 3.3 ~ 49.6% of neoaortic valve regurgitation [15, 16]. Many studies [15, 17–22] have reported various risk factors associated with the occurrence and progression of neoaortic valve regurgitation, such as the presence of a VSD, bicuspid native pulmonary valve, primary pulmonary valve regurgitation, CA transplantation method, and higher neoaortic root/ascending aorta ratio. In the CRANR technique, we have to make one or two small incisions at the closed neoaorta adjacent neoaortic valve for CA transfer, which can cause postoperative neoaortic valve regurgitation. Thus, we have been using marking stitches around the anterior commissure of the MPA to avoid injury to the valve while making the incisions. We believe this maneuver is highly effective in preventing injury to the neoaortic valve. In addition, we are trying to put the CA button as high as possible to avoid the disruption of the sinotubular junction of the neoaorta. We evaluated the incidences of early and late postoperative neoaortic valve regurgitations, and in the CRANR group, the incidences of mild or more neoaortic valve regurgitation was 15.3% at discharge and the rate of the 5- and 10-year freedom from it was 66.1 and 61.6%, respectively. The incidences of early and late mild or more neoaortic valve regurgitation was not as high as compared with the OCR group. The degree of neoaortic valve regurgitation in our series was mostly mild. Only two patients from the CRANR group had moderate neoaortic valve regurgitation, but both are doing well without valve reintervention. We do not know why the two moderate neoaortic valve regurgitations developed only in the CRANR group.

### Study limitations

This study was limited by its retrospective nature. The OCR technique was performed by one surgeon, but the CRANR technique was performed by five surgeons. The modification of the coronary transfer technique was not the sole factor accounting for the improvement of the operative results because not all operative deaths were related to the coronary problem, even though most of them were related. There was a time-frame difference between the two groups. The CRANR technique was adopted when the surgeon already had enough experience in ASO. However, it is clear that the 30-day mortality has been dramatically reduced after adopting the CRANR technique.

## Conclusion

CA reimplantation after neoaortic reconstruction can reduce intraoperative or postoperative CA-related problems by allowing surgeons to more easily and more accurately perform coronary transfer in ASO without increasing neoaortc valve regurgitation.

## Data Availability

The datasets generated and analysed during the current study are not publicly available due to IRB permission. But if there are reasonable request, then we try to get permission about this and then available from the corresponding author.

## Abbreviations

ACC: Aortic cross clamp
ASO: Arterial switch operation
CA: Coronary artery
CPB: Cardiopulmonary bypass
CRANR: Coronary reimplantation after neoaortic reconstruction’
MPA: Main pulmonary artery
OCR: Open coronary reimplantation
TGA: Transposition of the great arteries
VSD: Ventricular septal defect
